# Supplementary figures and images for: Salt and osmotic stress can improve the editing efficiency of CRISPR/Cas9-mediated genome editing system in potato
Source: PeerJ. 2023 Jul 31;11:e15771. doi: 10.7717/peerj.15771 (PMC10399558; doi:10.7717/peerj.15771)

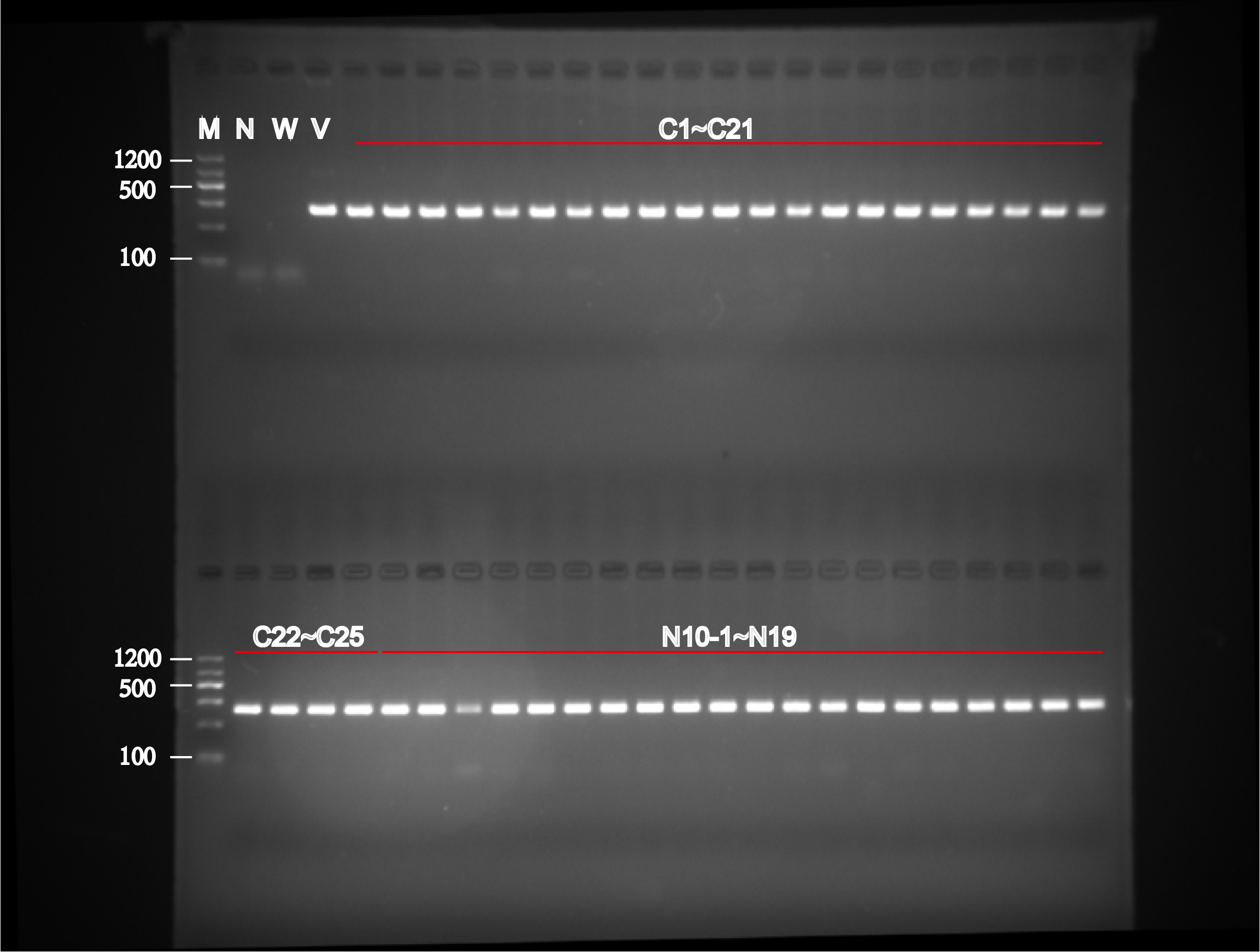

Supplement: Supplemental Information 3 [file peerj-11-15771-s003.zip › Figure 2 raw_data/1.png]

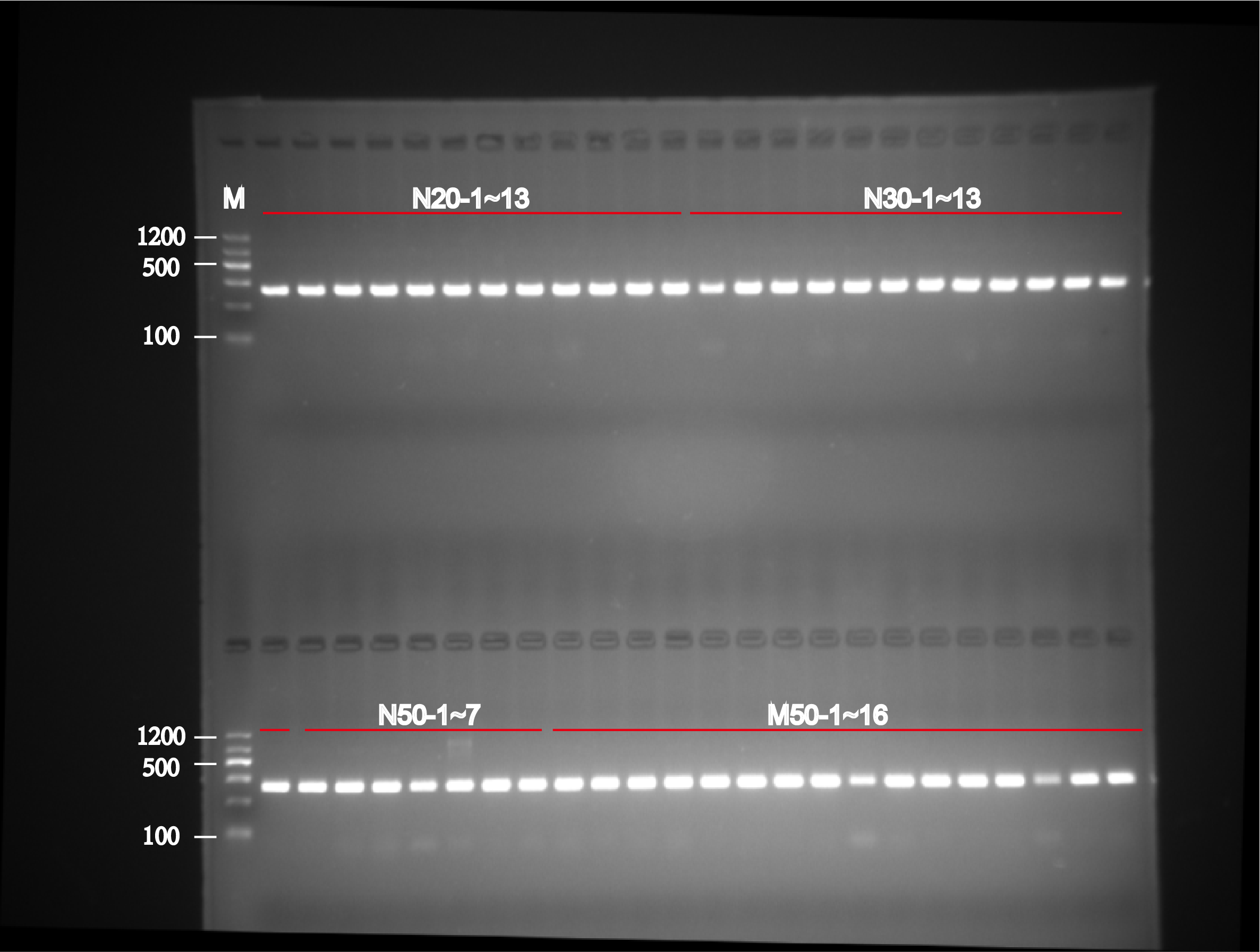

Supplement: Supplemental Information 3 [file peerj-11-15771-s003.zip › Figure 2 raw_data/2.png]

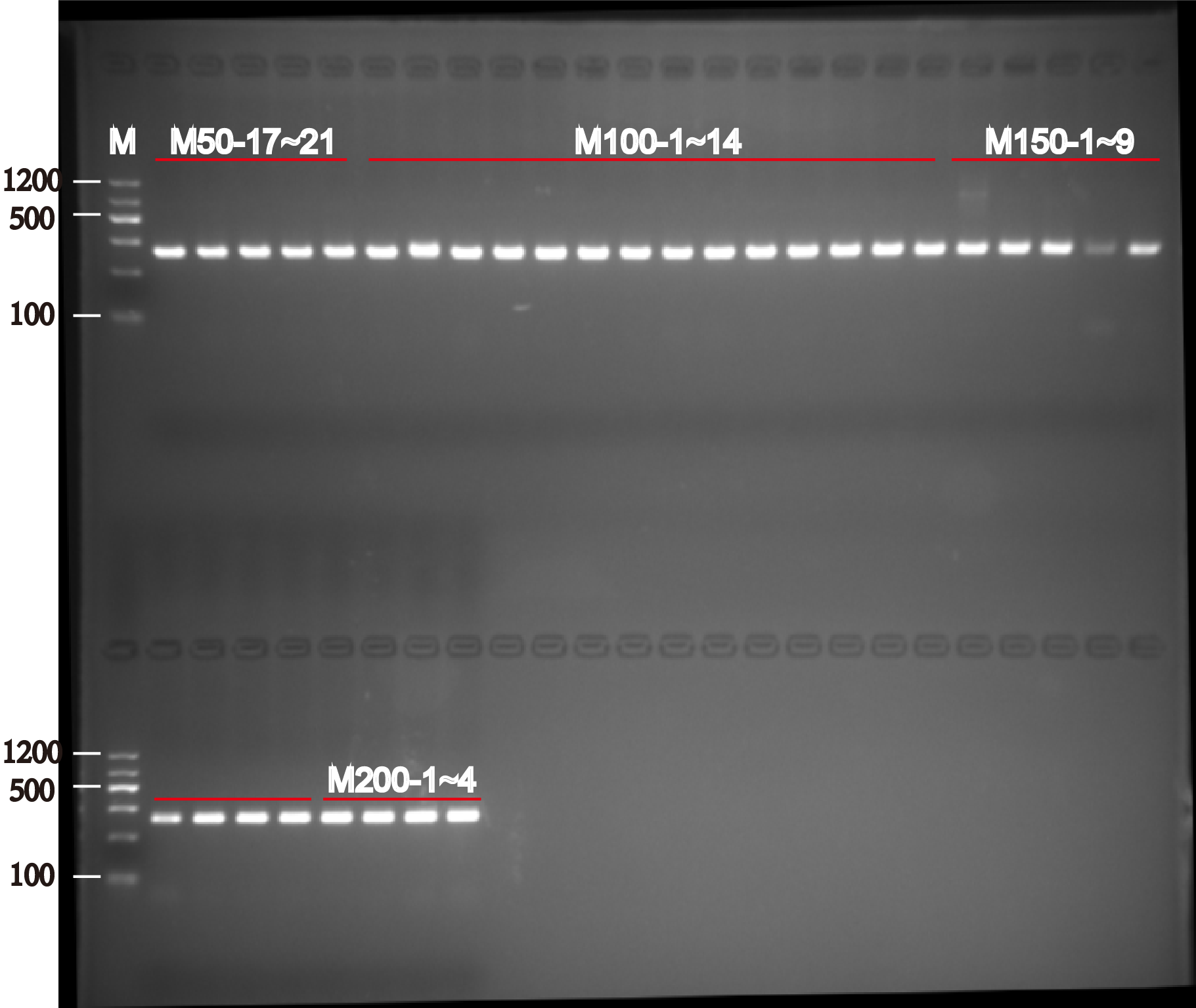

Supplement: Supplemental Information 3 [file peerj-11-15771-s003.zip › Figure 2 raw_data/3.png]
